# *Vibrio cholerae* SXT Element, Laos

**DOI:** 10.3201/eid1102.040794

**Published:** 2005-02

**Authors:** Claudia Toma, Noboru Nakasone, Tianyan Song, Masaaki Iwanaga

**Affiliations:** * University of the Ryukyus, Okinawa, Japan

**Keywords:** letter, Vibrio cholerae, SXT element, Laos

**To the Editor:** The SXT element is a *Vibrio cholerae*–derived ICE (integrating and conjugative element), which has also been referred to as a conjugative transposon (*1*) or a constin (*2*). ICEs excise from the chromosomes of their hosts, transfer to a new host through conjugation, and then integrate into the chromosome again. SXT element was originally isolated in 1993 from a *V. cholerae* O139 clinical isolate (SXT^MO10^) (1). The ≈100-kbp SXT element confers resistance to sulfamethoxazole, trimethoprim, chloramphenicol, and streptomycin (*1*). Since 1994, *V. cholerae* isolates from Bangladesh, India, and Mozambique have also contained the SXT element (*2**–**4*). In SXT^MO10^, resistance genes are embedded near the 5´ end, in a ≈17.2-kbp composite transposon-like element that interrupts the SXT-encoded *rumAB* operon. In contrast, in El Tor O1 *V. cholerae* strains isolated in India and Bangladesh, the resistance genes are located in SXT^ET^, which is closely related but not identical to SXT^MO10^ (*2*). Comparison of 2 related ICEs, SXT of *V. cholerae* and R391 of *Providencia rettgeri* (*5*), showed that the conserved backbone apparently contains 3 hot spots for insertions of additional DNA sequences: the first between *sO43* and *traL*, the second between *trA* and *sO54*, and the third between *sO73* and *traF*. R391 contains an intact *rumAB* operon and a transposon-associated kanamycin resistance gene located ≈3.5 kbp from the *rumAB* operon (*6*). Mobile genetic elements such as SXT have a crucial role in spreading antimicrobial drug resistance genes among microbial populations, and our understanding of these genetic elements would help to control the emergence of antimicrobial drug resistance.

We have been monitoring the drug sensitivity pattern in the Lao People's Democratic Republic (Laos) since 1993, and we have found that *V. cholerae* O1 strains isolated after 1997 were resistant to tetracycline, sulfamethoxazole, trimethoprim, chloramphenicol, and streptomycin (*7*). Analysis of the genetic determinants encoding antimicrobial drug resistance showed an SXT element (SXT^LAOS^), which is different from the previously reported SXTs (*8*). SXT^LAOS^ contains 2 novel open reading frames (ORFs) in the third hot spot (between *sO73* and *traF*). SXT^ET^ contains a class 9 integron in hot spot *sO73-traF* that harbors *dfrA1* as a gene cassette (*2*). In SXT^MO10^, the gene encoding trimethoprim resistance (*dfr18*) is encoded in the ≈17.2-kbp composite transposon-like element that interrupts the SXT-encoded *rumAB* operon. SXT^LAOS^ does not encode *dfr18* or *dfrA1*, and the gene encoding trimethoprim resistance has not been identified. In this study, we analyzed hot spot *sO43-traL* and hot spot *traA-sO54* to better characterize SXT^LAOS^.

Two sets of primers were designed to amplify the hot spot regions. Primer HS1-F, which anneals to *sO43*, was 5´ GGC TAT TCC ACC GGT GGT G 3´; primer HS1-R, which anneals to *traL*, was 5´ TGC CGA TCA CTA GCC CCA AC 3´; primer HS2-F, which anneals to *traA*, was 5´ ATG GGT CTC TAC AAT ACG CC 3´; and primer HS2-R, which anneals to *sO54*, was 5´ GGA GAC AGC GCA AGC GCC AG 3´. Polymerase chain reaction (PCR) amplifications on genomic DNA extracted from the *V. cholerae* O1 strain isolated in Laos (strain 00LA1) with primers HS1-F and HS1-R yielded an amplicon of ≈1100 bp, which is slightly different from the amplicon obtained with DNA extracted from *V. cholerae* O139, strain MO10 (≈1,000 bp). PCR amplification using primers HS2-F and HS2-R gave amplicons of similar size (≈2,200 bp) for both strains. The ≈1,000-bp and ≈2,200-bp PCR products from strain 00LA1 were cloned independently into the pCR 2.1 vector and tested to determine if recombinant plasmids confer trimethoprim resistance after transformation to *Escherichia coli*. No trimethoprim-resistant colonies were observed after transformation. The nucleotide sequences of the inserted fragments were analyzed. The region between *sO43* and *traL* showed 97% identity to the corresponding region of *P. rettgeri* R391 (accession no. AY090559), which encodes 2 hypothetical proteins (ORF 37 and ORF 38). The region between *traA* and *sO54* showed 97% identity to the corresponding region of SXT^MO10^ (accession no. AY055428). Since the gene encoding trimethoprim resistance was not located in any of the hot spot regions proposed by Beaber et al. (*5*), we also analyzed the region between *sO26* and *sO27*, which in R391 contains the kanamycin resistance gene. Primers sO26-F (5´ GAG CAA TGG GCG AGA GTT CC) and s027-R (5´ TCA GCG ACA ACC GGA GAA TG) gave an amplicon of 409 bp for SXT^MO10^, as expected, while no PCR product was obtained for SXT^LAOS^. This result suggested that the region between *sO26* and *sO27* in SXT^LAOS^ is also different from SXT^MO10^.

*V. cholerae* O139 has not been isolated in Laos, and the SXT element was not likely transmitted from a *V. cholerae* O139 strain to a *V. cholerae* O1 strain. Since SXT^LAOS^ has a hot spot that is identical to R391, we show evidence for a possible independent emerging of SXT^LAOS^. Further analysis is needed to understand the evolution and relationship between different ICEs and the emergence of new variants.

In a previous study (*8*), we confirmed experimentally that trimethoprim resistance was also transferred by conjugation, and we hypothesized that the responsible gene is located within SXT^LAOS^. However, the gene was not found in any of the proposed hot spot regions. The possibility that the trimethoprim resistance determinant is located on the chromosome outside the SXT element and cotransfers with the SXT in an Hfr-like manner cannot be ruled out (*9*). Therefore, additional hot spot regions may exist in SXT elements for insertion of DNA; otherwise the trimethoprim resistance gene is not encoded within SXT^LAOS^.

The nucleotide sequence data reported in this study will appear in the DDBJ/EMBL/GenBank nucleotide sequence databases with the accession numbers AB185252 for the hot spot *sO43-traL* and AB186353 for the hot spot *traA-sO54*.

## References

[1] Waldor MK, Tschäpe H, Mekalanos JJ. A new type of conjugative transposon encodes resistance to sulfamethoxazole, trimethoprim, and streptomycin in *Vibrio cholerae* O139. J Bacteriol. 1996;178:4157–65. 8763944 10.1128/jb.178.14.4157-4165.1996 PMC178173

[2] Hochhut B, Lotfi Y, Mazel D, Faruque SM, Woodgate R, Waldor MK. Molecular analysis of antibiotic resistance gene clusters in *Vibrio cholerae* O139 and O1 SXT constins. Antimicrob Agents Chemother. 2001;45:2991–3000. 10.1128/AAC.45.11.2991-3000.2001 11600347 PMC90773

[3] Amita C Sr. Thungapathra M, Ramamurthy T, Nair GB, Ghosh A. Class I integrons and SXT elements in El Tor strains isolated before and after 1992 *Vibrio cholerae* O139 outbreak, Calcutta, India. Emerg Infect Dis. 2003;9:500–2. 12702236 10.3201/eid0904.020317 PMC2957977

[4] Dalsgaard A, Forslund A, Sandvang D, Arntzen L, Keddy K. *Vibrio cholerae* O1 outbreak in Mozambique and South Africa in 1998 are multiple-drug resistant, contain the SXT element and the *aadA2* gene located on class 1 integrons. J Antimicrob Chemother. 2001;48:827–38. 10.1093/jac/48.6.827 11733467

[5] Beaber JW, Burrus V, Hochhut B, Waldor MK. Comparison of SXT and R391, two conjugative integrating elements: definition of a genetic backbone for the mobilization of resistance determinants. Cell Mol Life Sci. 2002;59:2065–70. 10.1007/s000180200006 12568332 PMC11146103

[6] Böltner D, Mac Mahon C, Pembroke JT, Strike P, Osborn A. R391: a conjugative integrating mosaic comprised of phage, plasmid, and transposon elements. J Bacteriol. 2002;184:5158–69. 10.1128/JB.184.18.5158-5169.2002 12193633 PMC135318

[7] Phantouamath B, Sithivong N, Sisavath L, Munnalath K, Khampheng C, Insisiengmay S, Transition of drug susceptibilities of *Vibrio cholerae* O1 in Lao People's Democratic Republic. Southeast Asian J Trop Med Public Health. 2001;32:95–9. 11485103

[8] Iwanaga M, Toma C, Miyazato T, Insisiengmay S, Nakasone N, Ehara M. Antibiotic resistance conferred by a class I integron and SXT constin in *Vibrio cholerae* strains isolated in Laos. Antimicrob Agents Chemother. 2004;48:2364–9. 10.1128/AAC.48.7.2364-2369.2004 15215082 PMC434172

[9] Hochuut B, Marrero J, Waldor MK. Mobilization of plasmids and chromosomal DNA mediated by the SXT element, a constin found in *Vibrio cholerae* O139. J Bacteriol. 2000;182:2043–7. 10.1128/JB.182.7.2043-2047.2000 10715015 PMC101929

